# Alterations in brain leptin signalling in spite of unchanged CSF leptin levels in Alzheimer’s disease

**DOI:** 10.1111/acel.12281

**Published:** 2014-12-02

**Authors:** Silvia Maioli, Maria Lodeiro, Paula Merino-Serrais, Farshad Falahati, Wasim Khan, Elena Puerta, Alina Codita, Roberto Rimondini, Maria J Ramirez, Andrew Simmons, Francisco Gil-Bea, Eric Westman, Angel Cedazo-Minguez, Alzheimer's Disease Neuroimaging Initiative

**Affiliations:** 1 Karolinska Institutet, Department of Neurobiology Care Sciences and Society, Center for Alzheimer Research, Division for NeurogeriatricsStockholm, Sweden; 2 Karolinska Institutet Department of Neurobiology Care Sciences and Society, Center for Alzheimer Research, Division for clinical geriatricsStockholm, Sweden; 3 Institute of Psychiatry, King’s College LondonLondon, UK; 4 Medical and Surgical Science, Department-DIMEC-University of BolognaBologna, Italy; 5 Department of Pharmacology and Toxicology, University of NavarraPamplona, Spain; 6 NIHR Biomedical Research, Centre for Mental Health, King’s College LondonLondon, UK; 7 NIHR Biomedical Research, Unit for Dementia, King’s College LondonLondon, UK; 8 Department of Cellular and Molecular Neuropharmacology, Division of Neurosciences, Center for Applied Medical Research (CIMA), University of NavarraPamplona, Spain

**Keywords:** Alzheimer’s disease, amyloid-beta, ApoE genotype, CSF, leptin receptors, leptin

## Abstract

Several studies support the relation between leptin and Alzheimer’s disease (AD). We show that leptin levels in CSF are unchanged as subjects progress to AD. However, in AD hippocampus, leptin signalling was decreased and leptin localization was shifted, being more abundant in reactive astrocytes and less in neurons. Similar translocation of leptin was found in brains from Tg2576 and apoE4 mice. Moreover, an enhancement of leptin receptors was found in hippocampus of young Tg2576 mice and in primary astrocytes and neurons treated with Aβ_1-42_. In contrast, old Tg2576 mice showed decreased leptin receptors levels. Similar findings to those seen in Tg2576 mice were found in apoE4, but not in apoE3 mice. These results suggest that leptin levels are intact, but leptin signalling is impaired in AD. Thus, Aβ accumulation and apoE4 genotype result in a transient enhancement of leptin signalling that might lead to a leptin resistance state over time.

## Introduction

The hormone leptin is mainly synthesized by adipocytes and circulates in the plasma in proportion to fat mass (Klein *et al*., 1996). Leptin acts as a regulator of body energy expenditure and food intake (Elmquist *et al*., 1998). Circulating leptin is taken into the brain, across the blood brain barrier (BBB), where its main action is in the hypothalamus. However, leptin receptors (LepR) are widely expressed in other brains regions including the hippocampus. Accumulating evidence from cell and animal models shows positive effects on memory processes and neuroprotection (Shanley *et al*., 2001; Harvey *et al*., 2006; Guo *et al*., 2008). In addition, leptin presents various functions in the immune system, including inflammation (Paz-Filho *et al*., 2013), and plays a major role in the chronic pro-inflammatory state that is seen in obesity and atherosclerosis (Conde *et al*., 2011).

An increasing number of epidemiological and experimental studies provide support for the link between leptin and Alzheimer’s disease (AD) pathogenesis. Circulating levels of leptin were reported to be significantly lower in patients with AD as compared to controls (Power *et al*., 2001). Recently, Lieb *et al*. reported that high plasma concentrations of leptin correlate with a significantly lower risk of AD, larger cerebral brain and hippocampal volumes (Lieb *et al*., 2009). In contrast, Rajagopalan *et al*. showed that high leptin levels in plasma correlate with volume loss in several brain regions, regardless of clinical diagnosis or body mass index (BMI) (Rajagopalan *et al*., 2012). Another recent study reported significant elevation in leptin levels in both CSF and hippocampus of patients with AD (Bonda *et al*., 2013). The importance of leptin in AD is suggested by several studies. *In vitro*, leptin treatment is reported to reduce Aβ levels in neurons by inhibiting β-secretase activity and to reduce tau phosphorylation through the modulation of tau kinases (Greco *et al*., 2008, 2009). Leptin treatment of murine models of AD resulted in significant reductions of Aβ, phosphorylated Tau (p-tau) and cognitive deficits (Greco *et al*., 2010). Based on this, the possibility of a potential use of leptin as a replacement therapy in the treatment of AD has been proposed.

The aims of this study were to (A) clarify whether brain leptin levels are affected during the progression of AD, (B) investigate the relationship between CSF leptin levels and hippocampal volume, AD biomarkers, such as Aβ, total-tau (t-tau), phospho-tau (p-tau) and inflammatory markers. In addition, the cellular localization and distribution of leptin and leptin receptors (LepR) were studied in hippocampus and frontal cortex from patients with AD and control individuals, as well as in mice models of relevance for AD pathology (Tg2576, apoE4 and apoE3). Finally, (C) the possible effects of Aβ on leptin and LepR signalling were also investigated in primary astrocyte and neuronal cell cultures.

## Results

### CSF leptin levels in the ADNI cohort study

The results of one-way ANOVA analysis for age, education, leptin levels, interleukin-6 receptors (IL-6R) and interleukin-3 (IL-3) in CSF revealed no significant differences between the four diagnostic groups (AD, MCI-c, MCI-s and CTL). The mean MMSE, hippocampal volume, Aβ_1-42_, t-tau and p-tau CSF levels were significantly different (*P* < 0.001) between the diagnostic groups (Table1).

**Table 1:** Study cohort

	Healthy controls *N* = 88	MCI stable *N* = 81	MCI converters *N* = 46	Alzheimer’s disease *N* = 63	ANOVA *P*-value
Gender (Female/Male)	42/46	27/54	18/28	28/35	
Age	75.8 ± 5.5	74.1 ± 7.0	75.3 ± 7.0	74.4 ± 7.6	0.369
Education	15.6 ± 3.0	16.2 ± 3.0	15.8 ± 2.7	15.1 ± 2.9	0.139
MMSE score	29.1 ± 1.0	27.1 ± 1.7	26.3 ± 1.8	23. 5 ± 1.8	<0.001
Nr. of APOE4 alleles (0/1/2)	66/20/2	41/31/9	16/21/9	18/30/15	
Hippocampal volume	0.00280 ± 0.00034	0.00237 ± 0.00037	0.00216 ± 0.00034	0.00219 ± 0.00041	<0.001
Leptin	17.0 ± 2.5	16.5 ± 2.5	16.1 ± 2.6	17.1 ± 2.4	0.128
IL6R	5.05 ± 0.14	5.06 ± 0.15	5.02 ± 0.14	5.03 ± 0.13	0.420
IL3	8.5 ± 1.8	8.4 ± 1.9	8.0 ± 2.0	7.9 ± 1.9	0.173
P-Tau	24.9 ± 13.2	34.7 ± 16.4	39.9 ± 15.2	43.3 ± 20.4	<0.001
Aβ_1-42_	206 ± 57	164 ± 55	146 ± 39	138 ± 31	<0.001
T-Tau	69 ± 28	101 ± 53	111 ± 50	130 ± 60	<0.001

Data are represented as mean ± standard deviation. Education and age given in years. MMSE = Mini Mental State Examination. Hippocampus volume is normalized to intracranial volume.

Table2 shows the results of bivariate correlation between pairs of leptin, hippocampal volume, Aβ_1-42_, t-tau, p-tau, IL-6R and IL-3 for the entire cohort and also for only CTL and MCI subjects. In the entire cohort, significant correlations were observed between leptin and hippocampal volume (Pearson coef. = 0.155, *P* = 0.009), between leptin and IL-6R (Pearson coef. = −0.121, *P* = 0.043), and between leptin and IL-3 (Pearson coef. = −0.125, *P* = 0.037). In particular, correlations between leptin and hippocampus were investigated with regard to age, education, gender and apoE4 genotype. A linear regression model was used to adjust for the effect of age, education, gender and apoE genotype (E4 carriers vs. noncarriers) on the correlation. Table3 shows the result of the linear regression model. After adjustments, the correlation between leptin and hippocampal volume was not significant. According to the regression model, the observed correlation between leptin and hippocampal volume was due to gender variations. Further testing by means of independent sample *t*-test highlighted that both leptin and hippocampal volume are significantly different between female (higher) and male (lower) subjects (see supplementary data, Table S2 and Figure S1).

**Table 2:** The results of bivariate correlation between CSF leptin, hippocampal voulme and other CSF biomarkers

	Leptin	Hippo campal volume	P-TAU	Aβ_1-42_	T-TAU	IL-6R	IL-3
Leptin		0.155 (0.009)	−0.084 (0.160)	0.051 (0.393)	−0.052 (0.392)	−0.121 (0.043)	−0.125 (0.037)
Hippocampal volume	*0.260 (<0.001)*		−0.243 (<0.001)	0.311 (<0.001)	−0.230 (<0.001)	−0.007 (0.910)	0.005 (0.940)
P-TAU	*−0.075 (0.272)*	*−0.245 (<0.001)*		−0.474 (<0.001)	0.771 (<0.001)	0.184 (0.002)	0.026 (0.669)
Aβ_1-42_	*0.076 (0.265)*	*0.271 (<0.001)*	*−0.504 (<0.001)*		−0.403 (<0.001)	0.068 (0.261)	0.212 (<0.001)
T-TAU	*−0.065 (0.342)*	*−0.198 (0.004)*	*0.759 (<0.001)*	*−0.409 (<0.001)*		0.261 (<0.001)	0.087 (0.147)
IL-6R	*−0.179 (0.008)*	*−0.013 (0.845)*	*0.233 (0.001)*	*0.040 (0.559)*	*0.277 (<0.001)*		0.413 (<0.001)
IL-3	*−0.061 (0.370)*	*−0.053 (0.442)*	*0.029 (0.669)*	*0.198 (0.004)*	*0.094 (0.172)*	*0.432 (<0.001)*	

The upper-right triangle represents the results of bivariate correlation using data from all subjects, and the lower-left triangle (italic values) represents the correlations using only HC and MCI subjects. Data are represented as Pearson correlation coefficients (*P*-value).

**Table 3:** Results of linear regression analyses for leptin and hippocampal volume

	Standardized coefficient	*P*-value
Hippocampal volume	0.024	0.676
Age (years)	−0.068	0.213
Education (years)	−0.094	0.084
Gender (0: female, 1: male)	−0.468	<0.001
APOE4 category (0: negative, 1: positive)	−0.009	0.874

Dependent variable is leptin and adjustment made for age, education, gender and APOE 4 category.

### Alterations in leptin and leptin signalling in AD brains

**Figure 1:**
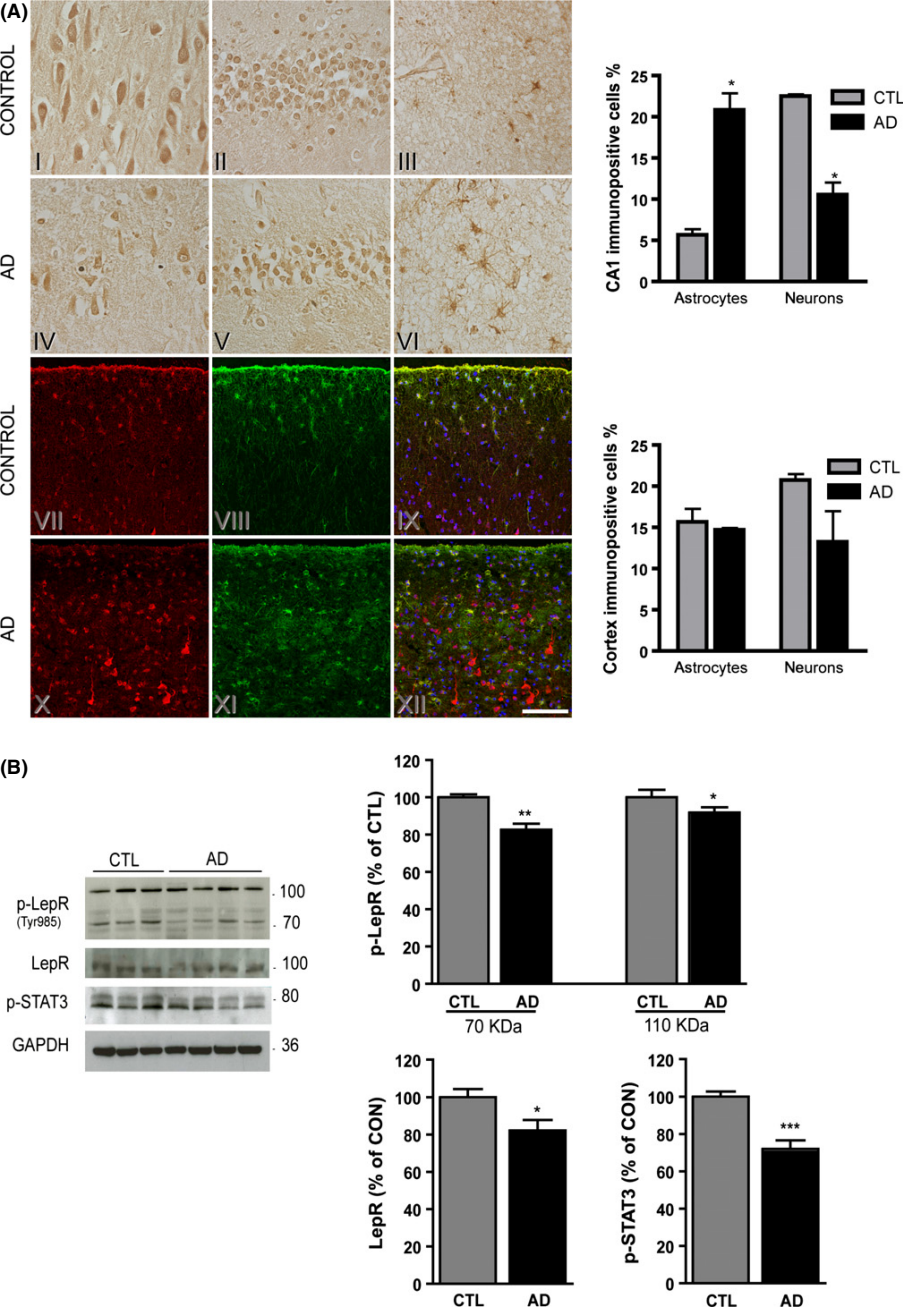
Alterations in leptin localization and leptin signalling in AD brains. (A) Leptin immunoreactivity in AD and control brains. (I–VI) Photomicrographs of sections DAB stained with leptin showing different fields of the hippocampal formation (I,IV, III, VI, CA1 region and II, V granular layer) from control (I–III) and patient with AD (IV–VI). (VII–XII) Confocal microscopy images of the frontal cortex from control (VII–IX) and patient with AD (X–XII) showing triple staining of leptin (red) and GFAP (green) antibodies and DAPI (blue). (VII–XII, X–XII) Stacks of 11 and 7 confocal optical sections, respectively (step size: 1 μm). Scale bar (in XII): 50 μm in I–VI; 120 μm in VII–XII. Quantification of astrocytes and neurons immunopositive for leptin in CA1 and frontal cortex of AD and control brains. Number of stained cells is given as percentage of total number of cells (*P < 0.05). (B) Decrease of leptin signalling in hippocampus of AD brains. Immunoblots from hippocampal homogenates from CTL and patients with AD, using anti-p-LepR, anti-LepR and anti-p-STAT3 antibodies. Data are shown as mean ± standard error of mean of immunoreactivity (OD × area of the band) normalized by GAPDH levels. CTL were used as reference. (*P < 0.05, **P < 0.01, ***P < 0.001, *N* = 4).

Pyramidal neurons, granular neurons and reactive astrocytes were immunoreactive for leptin in hippocampus sections from controls and AD (Fig.1A, I–VI). In frontal cortex sections from the same individuals, a similar staining pattern of leptin in pyramidal neurons and astrocytes was observed (Fig.1A, VII–XII). In the hippocampus of control brains, the number of neurons immunopositive for leptin was significantly higher than in AD brains (*P* = 0.015). In contrast, in the hippocampus of AD brains, leptin staining was mainly present in reactive astrocytes, being the number of reactive astrocytes immunopositive for leptin significantly higher than controls (*P* = 0.028). In cortex, no differences were found between AD and controls; however, in AD cortex, high leptin signal inside some neurons, morphologically resembling tau-bearing or ‘ghost’ neurons (Fig.1A, X), was seen. Double immunostaining leptin/p-tau experiments revealed that this was not the case (data not shown).

Levels of p-LepR (activated LepR), LepR and p-STAT3 (as additional indicator of LepR activity) were analysed in hippocampal samples by immunoblotting. As shown in Fig.1B, the immunoblotting pattern of p-LepR showed two major bands (at approximately 110 and 70 kDa), corresponding with the long and the short isoforms of LepR (Ebenbichler *et al*., 2002). The immunoreactivity of both p-LepR forms was decreased in AD brains compared to controls (*P* = 0.008 and *P* = 0.03, respectively, for 70 and 110 kDa). LepR and p-STAT3 levels were also significantly decreased in hippocampus of AD subjects when compared to controls (Fig.1B; *P* = 0.01, *P* = 0.0004, respectively).

### Changes in leptin localization and leptin receptor expression in the brain of Tg2576 and apoE4 mice

**Figure 2:**
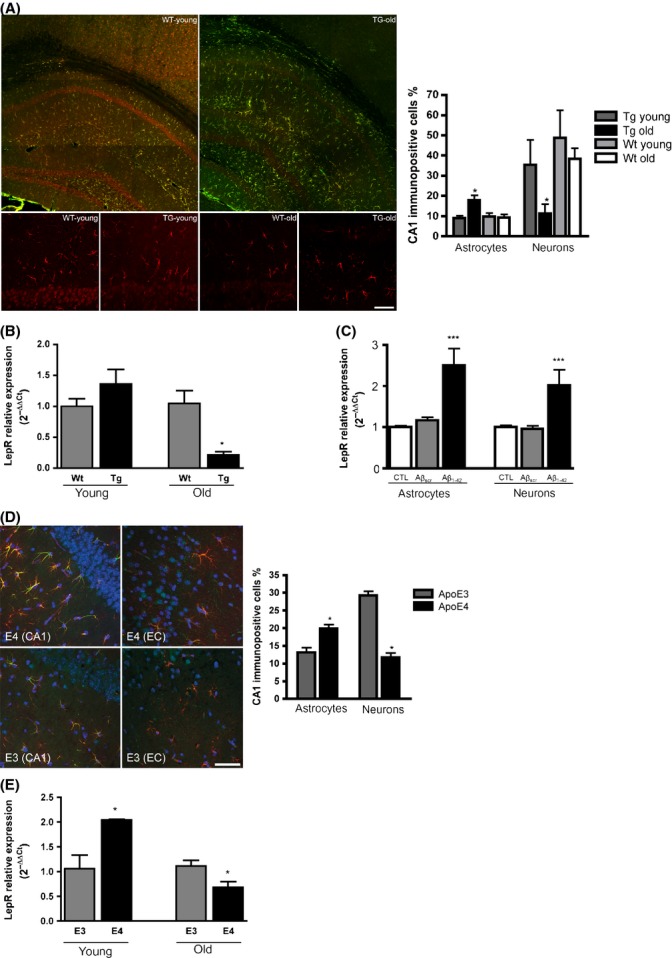
Alterations in leptin localization and leptin receptor expression in Tg2576 and apoE4 mice. (A) Leptin immunoreactivity in the brain of wild-type and Tg2576 mice. Upper images: confocal microscopy field view of a hippocampus from young (7-month-old) Wt and old (15-month-old) Tg2576, showing the cellular distribution of anti-leptin antibody (red) and anti-GFAP antibody (green). Lower images: representative images of CA1 region from young and old Wt and Tg2576 mice, respectively, showing leptin immunoreactivity (red) in pyramidal neurons and astrocytes. Scale bars for the upper images 180 μm; for the lower images 50 μm. Quantification of astrocytes and neurons immunopositive for leptin in the hippocampus of wild-type and Tg 2576. Number of stained cells is given as percentage of total number of cells (*P < 0.05, *N* = 4). (B) Altered LepR expression levels in hippocampus of Tg2576 mice. LepR expression levels were analysed by real-time RT-PCR in hippocampus from young (5-month-old) and old (24-month-old) Wt and Tg2576 mice. Data are presented as mean ± standard error of mean. (*P < 0.05, *N* = 4). (C) Aβ_1-42_ increases LepR expression levels in both astrocytes and neurons. (A) Rat primary astrocytes and hippocampal neuronal cultures were treated with Aβ_1-42_ and Aβ_scr_ (0.5 μm) for 24 h. LepR expression levels were analysed by real-time RT–PCR. Data are presented as mean ± standard error of mean (***, *P* < 0.001). Three independent experiments, each in triplicate, were performed.(D) Leptin immunoreactivity in apoE4 and apoE3 mice brain sections. Representative images of CA1 region and layer I-III of enthorinal cortex (EC) from apoE4 and apoE3 mice showing triple staining of leptin (green) and GFAP (red, as glial marker) antibodies and DAPI (blue, as nuclear marker). Scale bar = 50 μm. Quantification of astrocytes and neurons immunopositive for leptin in the hippocampus of apoE3 and apoE4. Number of stained cells is given as percentage of total number of cells (*P < 0.05, *N* = 4). (E) Altered LepR expression levels in hippocampus of apoE4 mice. LepR expression levels were analysed by RT–PCR in hippocampus from young (6-month-old) and old (16-month-old) apoE4 and apoE3 mice. Data are presented as mean ± standard error of mean. (*, *P* < 0.05, *N* = 4).

Immunofluorescence for leptin was performed in brains of young and old Tg2576 mice. Neurons and reactive astrocytes were immunoreactive for leptin in hippocampal sections from Wt and Tg2576 (Fig.2A). In old Tg2576 animals, leptin immunoreactivity was significantly decreased in neurons as compared to Wt old animals (*P* = 0.018). An increase in the number of astrocytes immunopositive for leptin was also found in old transgenic animals as compared to Wt old animals and to Tg young animals (*P* = 0.026) (Fig.2A).

We analysed the expression of LepR in Tg2576 mice hippocampus by RT–PCR. As shown in Fig.2B, a significant decrease in LepR expression in old Tg2576 mice compared to age-matched controls (*P* = 0.05) was found. Further, a trend was observed towards an increase in LepR expression in young Tg2576 mice compared to Wt animals.

We further investigated the possibility that Aβ_1-42_ could induce leptin expression or alter the expression of LepR in brain cells. No expression of leptin was found in brain samples from Wt and Tg2576 mice or in astrocyte and hippocampal neuronal rat primary cultures even after Aβ_1-42_ treatment. As shown in Fig.2C, a significant increase in LepR expression was found after 24 h of 0.5 μm Aβ_1-42_ treatment in both astrocytes and hippocampal neurons as compared to controls (CTL) but no effect of beta amyloid 1-42 scrambled (Aβ_scr_) on LepR expression was observed (Fig.2C; *P* < 0.001 CTL vs Aβ_1-42_, *P *< 0.001 Aβ_scr_ vs Aβ_1-42_ for both neurons and astrocytes).

The above results suggest that changes in leptin or leptin signalling observed in AD and Tg2576 mice brains might be mainly due to Aβ accumulation. Thus, another animal model of relevance for AD but without Aβ overproduction was studied (human apoE4 and apoE3 target replacement mice). ApoE4 mice show cognitive impairment (Grootendorst *et al*., 2005), and the apoE4 genotype is the strongest genetic risk factor for developing AD (Cedazo-Minguez, 2007). As seen in Fig.2D, neurons and reactive astrocytes were immunoreactive for leptin in hippocampus (CA1) and enthorinal cortex (EC) from both apoE3 and apoE4 mice. In apoE4 mice, the number of astrocytes immunopositive for leptin was significantly higher as compared to apoE3 mice (*P* = 0.056). In contrast, the number of neurons immunopositive for leptin was significantly decreased as compared to apoE3 (*P* = 0.012). When LepR expression was analysed in the hippocampus of ApoE4 mice, a similar result to Tg2576 mice was observed: in apoE4 mice, LepR expression was significantly increased in young animals and decreased in old animals, as compared to ApoE3 mice (Fig.2E, *P* = 0.02 for young mice; *P* = 0.04 for old mice). No expression of leptin was found in brain tissue from apoE3 and apoE4 mice (data not shown).

## Discussion

Several recent studies have focused on the involvement of leptin in AD, but its role in the pathological processes is still not clear. Until now, studies on the association between circulating leptin levels and AD have shown opposite and paradoxical results. In a small case–control study, leptin levels were observed to be low in patients with AD (Power *et al*., 2001). Lieb *et al*. have reported that in normal individuals, elevated plasma leptin levels are associated with lower incidence of dementia, AD and larger brain volumes (Lieb *et al*., 2009). On the other hand, Rajagopalan *et al*. reported that higher leptin plasma levels correlated with lower brain volumes (Rajagopalan *et al*., 2012). Moreover, circulating leptin may not adequately represent CSF leptin levels (Schwartz *et al*., 1996), and, to our knowledge, the levels of leptin in CSF and its association with AD pathology and progression have been investigated only in a small cohort (Bonda *et al*., 2013).

In this study, we measured leptin levels in the CSF of 278 subjects from the ADNI cohort. The leptin levels were correlated with the main CSF biomarkers for AD, such as Aβ_1-42_, p-tau or t-tau. As an association between plasma leptin and hippocampal volume has been reported (Lieb *et al*., 2009), we also explored the possibility of a relationship between CSF leptin levels and hippocampal volume, independently of per cent body fat. Noteworthy, our data showed no significant changes in CSF leptin levels among controls, MCI-s, MCI-c and patients with AD. In a linear regression analysis across all individuals, adjusting for age, gender, diagnostic groups, MMSE, education and apoE genotype, the correlation between hippocampal volume and leptin was found to be due to differences in gender. CSF leptin levels were significantly higher in women than in men. This difference observed is probably due to gender differences in body fat, sex hormones and insulin levels (Ostlund *et al*., 1996; Rosenbaum *et al*., 1996). When women and men were taken separately, no correlation was found between leptin and hippocampal volumes. Moreover, no correlation between leptin and CSF biomarkers of AD was found.

As leptin regulates and triggers inflammation by means of actions on its receptors and evidence suggest an involvement of leptin in the upregulation of inflammatory cytokines [for review see (Paz-Filho *et al*., 2013)], correlations between leptin and several inflammatory markers levels were also measured. Among all the cytokines available in this study, only IL-6R and IL-3 were found to correlate with leptin, although these correlations disappeared after adjustment for age, gender, diagnostic groups, MMSE, education and apoE genotype.

To better elucidate the cellular distribution of leptin in cerebral areas involved in the disease, immunohistochemical analyses of hippocampus and frontal cortex from patients with AD and controls were performed. In control individuals, leptin staining was mainly located in neurons; while in patients with AD, it was mainly present in reactive astrocytes. This pattern was seen in hippocampus but not in frontal cortex areas. Young and old Tg2576 mice were also analysed; the choice of different ages reflects two stages of AD pathology, because amyloid plaques and memory deficits are observed by 9 months of age in these mice. In old Tg2576 mice brains, leptin was reduced in neurons when compared to Wt old animals. Noteworthy, an increase of leptin staining in astrocytes was also found in old Tg2576 mice, suggesting that Aβ overproduction induces a decrease of leptin in neurons and a relocalization of leptin towards reactive astrocytes.

We next investigated the possibility of a direct effect of Aβ_1-42_ on leptin production and leptin signalling in the brain. Thus, leptin and LepR expression levels were measured in primary cultures of astrocytes and hippocampal neurons treated with Aβ_1-42_ and in the hippocampus of Tg2576 mice. Although there is some evidence for expression of leptin mRNA in the brain (Morash *et al*., 1999) and in a neuroblastoma cell line (Marwarha *et al*., 2011), we could not detect leptin expression in mice brain (wt, Tg2576, apoE3 or apoE4), neither in astrocytes nor hippocampal neurons from rat primary cultures, both in basal conditions or after treatment with Aβ_1-42_. Our results support the idea that leptin detected in the brain comes from the periphery. Thus, leptin transport at the BBB, BBB integrity and LepR levels in brain cells would be key factors for leptin levels and leptin signalling in brain. Leptin is transported across the BBB by a saturable and unidirectional system (Hileman *et al*., 2002), and impairment of leptin transport across the BBB has been reported in obesity (Burguera *et al*., 2000), contributing to a leptin-resistant state (Caro *et al*., 1996). This impairment has not been reported in AD, although it is suggested that the existence of a general BBB impairment in the late and severe stages of the disease (Viggars *et al*., 2011) would favour the permeability of leptin to the brain. However, this may not be the case in early stages of AD, because differences in CSF leptin levels at the early phases of AD progression were not found. At these early phases, BBB integrity remains intact, as demonstrated in a subgroup of CSF samples using albumin CSF/serum ratio (Supplementary Table S3). Noteworthy, AD brains used for immunohistochemistry and immunoblotting analyses were from subjects with severe AD, which would result in a more severe BBB impairment and consequently in increased leptin levels in the brain.

We found that Aβ_1-42_ treatment stimulates LepR synthesis in both astrocytes and neurons, suggesting that leptin signalling might be enhanced under Aβ accumulation conditions. This result was further confirmed in young Tg2576 mice, where an increase of LepR expression was found in the hippocampus compared to Wt. This is in agreement with previous work showing increased pSTAT3 levels in APP/PS1 transgenic mice before plaque formation (Wan *et al*., 2010). Treatment with leptin has been shown to reduce plaque burden in young Tg2576 mice (Fewlass *et al*., 2004). At that age, prior to plaque formation, Tg2576 animals already show BBB impairment (Ujiie *et al*., 2003), which could facilitate leptin delivery into the brain. According to our findings, these animals also have enhanced expression of LepR, which could further contribute to the positive effects of leptin treatment. However, in old Tg2576 mice, an opposite pattern with a significant decrease in LepR expression was observed. This suggests the existence of a downregulation of the signalling towards leptin resistance, which would question the effectiveness of leptin therapy at that stage of the pathology. In support of this, it has been reported that increased food intake causes obesity and insulin resistance in these animals (Kohjima *et al*., 2010). It has also been shown that acute treatment with Aβ_1-42_ in hippocampal neurons activates, while chronic treatment inhibits, STAT3 (Chiba *et al*., 2009). Importantly, we found a decrease in pSTAT3 in hippocampus of severe patients with AD, which is also in agreement with a previous report (Chiba *et al*., 2009). Based on this evidence, it might be suggested that Aβ_1-42_ initially causes an enhancement of leptin signalling, leading to leptin resistance over time.

Interestingly, apoE4 mice showed similar changes in leptin localization and LepR synthesis to those seen in Tg2576 mice. In apoE4 mice, leptin was also decrease in neurons and also more abundant in reactive astrocytes, and LepR expression was increased in the hippocampus of young animals but decreased in old animals, as compared to apoE3 mice. ApoE4 is a major risk factor for AD, but apoE4 mice do not show Aβ overproduction or plaque formation. Thus, a similar impairment of leptin signalling is occurring independently of Aβ, in an alternative animal model of relevance for AD pathology. As Tg2576 mice, apoE4 mice have BBB disruption at the age of 9 months (Bell *et al*., 2012), and signs of BBB impairment have been shown in humans carrying the apoE4 allele (Salloway *et al*., 2002). However, no reports have shown an association between apoE4 and leptin resistance in humans.

The mechanisms by which Aβ and apoE4 affect LepR expression are unknown. Both molecules have been shown to cause inflammatory responses, and LepR was shown to be upregulated in inflammatory conditions, such as LPS and TNFα treatments (Hsuchou *et al*., 2009). In fact, animal models for human Aβ overproduction or human apoE4 expression showed enhanced inflammatory reactions in the brain including TNFα generation and gliosis (Munch *et al*., 2003; Zhu *et al*., 2012). Thus, it may be speculated that the initial upregulation of LepR could result from the pro-inflammatory effects of Aβ or apoE4. On the other hand, chronic inflammation is known to cause leptin resistance (Martin *et al*., 2008).

In summary, no significant changes were observed in CSF leptin levels in AD, at least in stages where the BBB remains intact, as compared to controls. Animal models of Aβ accumulation and apoE4 genotype show a biphasic alteration in leptin signalling, with an early activation followed by a downregulation of leptin receptors. This downregulation is also seen in brains from individuals suffering of severe AD. These results suggest that alteration in leptin signalling should be considered when studying the putative neuroprotective effects of leptin in the disease. It is likely that the beneficial effects of leptin would be limited to early, rather than to late phases of AD.

## Experimental procedures

### Subjects

The study cohort includes 278 subjects: 88 controls (CTL), 63 subjects with AD, 81 subjects with stable mild cognitive impairment (MCI-s) and 46 MCI subject that converted to AD within 18 month after baseline (MCI-c) from the North American multicenter Alzheimer’s Disease Neuroimaging Initiative (ADNI). Additional information about the ADNI cohort, including inclusion criteria, can be found as supplementary data.

### Magnetic resonance imaging (MRI) and cerebrospinal fluid (CSF)

Both MRI and CSF data were downloaded from the ADNI website (www.loni.ucla.edu/ADNI). The description of CSF collection and data acquisition of the ADNI study can be found as supplementary data. MRI data from 1.5 T scanners were used with data collected from a variety of MR systems with protocols optimized for each type of scanner. The MRI protocol included a high-resolution sagittal 3D T1-weighted MPRAGE volume (voxel size 1.1 × 1.1 × 1.2 mm^3^) acquired using a custom pulse sequence specifically designed for the ADNI study to ensure compatibility across scanners. Full brain and skull coverage was required for the MRI data sets and detailed quality control carried out on all MR images according to previously published quality control criteria (Simmons *et al*., 2011).

### Hippocampal segmentation

Volumetric segmentation of the hippocampus was performed using the freesurfer software package, version 5.1.0 (http://surfer.nmr.mgh.harvard.edu/) (Fischl *et al*., 2002). The procedure automatically assigns a neuroanatomical label to each voxel in an MRI volume based on probabilistic information automatically estimated from a manually labelled training set. All hippocampal volumes (left and right side averaged) from each subject were normalized by the subject’s intracranial volume (Westman *et al*., 2013), which is estimated based on an affine transform in FreeSurfer. This segmentation approach has been used for multivariate classification of Alzheimer’s disease and healthy controls (Westman *et al*., 2011) and biomarker discovery (Thambisetty *et al*., 2010).

### Brain tissue, immunohistochemistry and immunoblotting

Brain material was obtained from the Brain Bank at Karolinska Institutet (Sweden). Immunohistochemistry was performed on hippocampus and frontal cortex from 4 brains from patients with definite AD (two males and two females 75–86-year-old) and 4 aged-matched controls (two females and two males, 66–87-year-old). For immunoblotting, 4 additional hippocampal samples from AD brains (males, 74–98-year-old) and 3 controls (males, 56–71-year-old) were used. DAB immunostaining, immunofluorescence and immunoblotting were performed as described previously (Akterin *et al*., 2006; Maioli *et al*., 2012). The list of primary antibodies is in supplementary Table1 (Table S1). Estimation of the number of cells immunopositive for leptin was performed using neurolucida neuron Tracing Software (MBF Bioscience). Three slides per mice and human brain were analysed, and number of stained cells is given as percentage of total number of cells.

### Transgenic mice

Five-month-old (young) and 2-year-old (old) female transgenic mice (Tg2576) overexpressing human amyloid precursor protein carrying the Swedish mutation (K670N/M671L) under the genetic mixed hybrid background C57BL/6/SJL and wild-type littermates (Wt) were used. Six-month-old (young) and 16-month-old (old) male hApoE Target Replacement (TR) mice expressing human apoE3 and apoE4, under the control of the murine apoE regulatory sequences and on a C57BL/6J background were used (Maioli *et al*., 2012). Four animals per genotype and age were used. All mice were kept under controlled temperature (21 °C ± 1) and humidity (55 ± 5%) on a 12-h light–dark cycle, and food/water was provided *ad libitum*. Experimental procedures were conducted in accordance with the European regulation and approved by the Ethical Committees of University of Navarra and Bologna.

### Rat primary culture and real-time RT–PCR

Cerebellar and hippocampal tissue from 16-day-old Sprague–Dawley rat embryos was homogenized in serum-free NeuroBasal medium with B27 supplement (2%). Astrocytes were serum-starved for 24 h before treatment. Aβ_1-42_ (Sigma-Aldrich, MO, USA) and Aβ_1-42_ scrambled (Aβ_scr_) (rPeptide, GA, USA) were prepared as previously described (Chiba *et al*., 2009) and used at 0.5 μm for 24 h. Ethical consent was received from the regional ethical committee of Karolinska Institutet. RNA extraction and real-time RT–PCR were performed as previously described (Mateos *et al*., 2011), using PCR Master Mix and TaqMan Gene Expression Assays (for rats: LepR Rn01433205_m1, GAPDH Rn01775763_g1; for mice, LepR Mm00440181_m1, GAPDH Catalogue Number 4352932E) (Life Technologies, CA, USA).

### Statistical analysis

One-way ANOVA was used to compare differences between mean levels of variables in the four groups, followed by HSD Tukey’s *post hoc* analysis. Bivariate correlation test was performed to calculate the correlation between pairs of variables, and Pearson correlation coefficient was reported. Linear regression analysis was utilized to calculate the adjusted correlation for covariates/confounders variables. Mann–Whitney test was used for immunoblotting. For quantification of the immunohistochemistry, Mann–Whitney and Kruskall–Wallis test were used. Unpaired *t*-test or one-way ANOVA followed by HSD Tukey’s *post hoc* analyses was used for RT–PCR experiments. A *P*-value < 0.05 was considered statistically significant.

## Supporting Information

**Data S1**ADNI cohort. **Table S1** List of primary antibodies used in immunohistochemistry (IH) and immunoblotting (IB). **Table S2** Mean comparison of Leptin and Hippocampal volume between male and female subjects. The results from independent sample *T*-test. **Table S3** No changes in the ratio Albumin in CSF/Albumin in serum, reflecting BBB integrity. **Figure S1** Mean value of Leptin levels of male and female subjects in different diagnostic groups. Error bars represent standard deviation.
