# Supplementary material for: Alterations in brain leptin signalling in spite of unchanged CSF leptin levels in Alzheimer’s disease
Source: Aging Cell. 2014 Dec 2;14(1):122–9. doi: 10.1111/acel.12281 (PMC4326905; doi:10.1111/acel.12281)
Supplement: Supplementary file 1 [file acel0014-0122-sd1.doc]

**Supplementary Data**

**ADNI cohort**

The ADNI was launched in 2003 by the National Institute on Aging (NIA), the National Institute of Biomedical Imaging and Bioengineering (NIBIB), the Food and Drug Administration (FDA), private pharmaceutical companies and non-profit organizations, as a 60 million, 5 year public-private partnership. The primary goal of ADNI has been to test whether serial magnetic resonance imaging (MRI), positron emission tomography (PET), other biological markers, and clinical and neuropsychological assessment can be combined to measure the progression of MCI and early AD. Determination of sensitive and specific markers of very early AD progression is intended to aid researchers and clinicians to develop new treatments and monitor their effectiveness, as well as lessen the time and cost of clinical trials.

The principal investigator of this initiative is Michael W. Weiner, MD, VA Medical Center and University of California – San Francisco. ADNI is the results of efforts of many co-investigators from a broad range of academic institutions and private corporations, and subjects have been recruited from over 50 sites across the U.S. and Canada. The initial goal of ADNI was to recruit 800 subjects but ADNI has been follow by ADNI-GO and ADNI-2. To date these three protocols have recruited over 1500 adults, ages 55 to 90, to participate in the research, consisting of cognitively normal older individuals, people with early or late MCI, and people with early AD.The follow up duration of each group is specified in the protocols for ADNI-1, ADNI-2 and ADNI-GO. Subjects originally recruited for ADNI-1 and ADNI-GO had the option to be followed in ADNI-2. For up-to-date information, see www.adni-info.org. The demographics of the cohort are given in Table 1. Inclusion criteria for the AD group were MMSE score between 20 and 26, CDR scale score of 0.5 or 1 and NINCDS/ADRDA criteria for probable AD. For inclusion in the MCI groups, criteria were a MMSE score between 24 and 30, memory problems with objective memory loss measured by the Wechsler Memory Scale Logical Memory II (education-adjusted scores), CDR score of 0.5, absence of significant levels of impairment in other cognitive domains, preservation of activities of daily living and absence of dementia. Inclusion criteria for the control group were MMSE score between 24 and 30, CDR score of 0, and absence of depression, MCI and dementia.

**CSF collection and data acquisition**

The description data acquisition of the ADNI study can be found at [www.loni.ucla.edu/ADNI/research/Cores/index.shtml](http://www.loni.ucla.edu/ADNI/research/Cores/index.shtml). CSF was collected in the morning after an overnight fast using a 20- or 24-gauge spinal needle, frozen within 1 hour of collection, and transported on dry ice to the ADNI Biomarker Core laboratory at the University of Pennsylvania Medical Center. The complete descriptions of the collection and transportation protocols are provided in the ADNI procedural manual at [www.adni-info.org](http://www.adni-info.org/).

Measurements of 76 analytes were excluded on the basis of quality control, leaving 83 analytes available for subsequent analysis. Distributions of data for individual analyses were checked for normality using Box-Cox methods and, when appropriate, log10 transformed to approximate a normal distribution.

**Acknowledgements**

ADNI is funded by the National Institute on Aging, the National Institute of Biomedical Imaging and Bioengineering, and through generous contributions from the following: Alzheimer’s Association; Alzheimer’s Drug Discovery Foundation; BioClinica, Inc.; Biogen Idec Inc.; Bristol-Myers Squibb Company; Eisai Inc.; Elan Pharmaceuticals, Inc.; Eli Lilly and Company; F. Hoffmann-La Roche Ltd and its affiliated company Genentech, Inc.; GE Healthcare; Innogenetics, N.V.; IXICO Ltd.; Janssen Alzheimer Immunotherapy Research & Development, LLC.; Johnson & Johnson Pharmaceutical Research & Development LLC.; Medpace, Inc.; Merck & Co., Inc.; Meso Scale Diagnostics, LLC.; NeuroRx Research; Novartis Pharmaceuticals Corporation; Pfizer Inc.; Piramal Imaging; Servier; Synarc Inc.; and Takeda Pharmaceutical Company. The Canadian Institutes of Health Research supports ADNI clinical sites in Canada. Private sector contributions are facilitated by the Foundation for the National Institutes of Health (www.fnih.org). The grantee organization is the Northern California Institute for Research and Education, and the study is coordinated by the Alzheimer's Disease Cooperative Study at the University of California, San Diego. ADNI data are disseminated by the Laboratory for Neuro Imaging at the University of California, Los Angeles. This research was also supported by NIH grants P30 AG010129 and K01 AG030514.

**Table s1.** List of primary antibodies used in immunohistochemistry (IH) and immunoblotting (IB)

| **anti-GFAP** | mouse | BD Bioscience | IH |
| --- | --- | --- | --- |
| **anti-leptin** | rabbit | Santa Cruz Biotechnology, USA | IH |
| **anti-LepR** | rabbit | Abcam, UK | IH |
| **anti-phospho-LepR(Tyr 985)** | rabbit | Santa Cruz Biotechnology, USA | IB |
| **anti-GADPH** | mouse | Enzo Life Sciences, US | IB |
| **STAT3** | rabbit | Cell Signalling Technology, USA | IB |

**Table s2.** Mean comparison of Leptin and Hippocampal volume between male and female subjects. The results from independent sample T-test.

|  | **Female** | **Male** | **P-value** |
| --- | --- | --- | --- |
| **Leptin** | 18.24 ± 1.95 | 15.67 ± 2.31 | <0.001 |
| **Hippocampal volume** | 0.00255 ± 0.00046 | 0.002357 ± 0.00042 | <0.001 |

**Figure s1.** Mean value of Leptin levels of male and female subjects in different diagnostic groups. Error bars represent standard deviation.


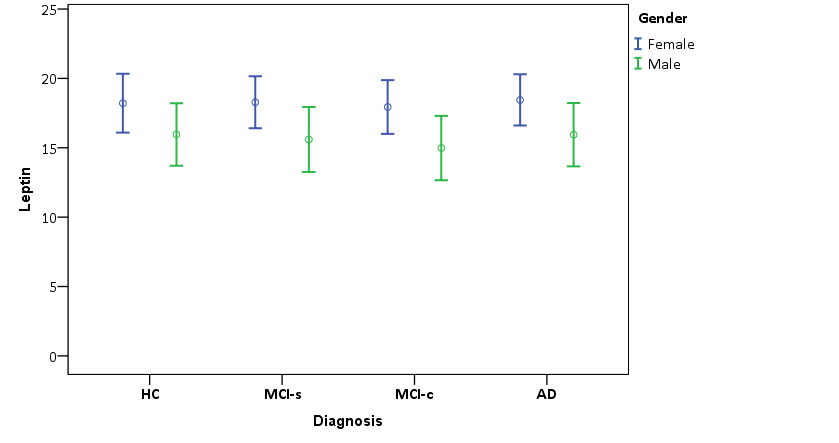


**Table s3.** No changes in the ratio Albumin in CSF/Albumin in serum, reflecting BBB integrity.

|  | **Healthy Control**  **N= 33** | **MCI Stable**  **MCI-Non converters**  **N = 26** | **MCI MCI-MCI-Converters**  **N=13** | **Alzheimer’s AD**  **N=27** | **ANOVA**  **P-value** |
| --- | --- | --- | --- | --- | --- |
| **Gender (Female/Male)** | 14/19 | 17/9 | 4/9 | 8/19 |  |
| **Age** | 57.5±1.1 | 61.6±2.0 | 63.1±2.3 | 68.6±1.8 |  |
| **Education** | 14 ±0.6 | 12.4 ±0.7 | 14±1.1 | 9.7±0.6 | <0.05 |
| **MMSE score** | 29.1±0.2 | 28.3±0.3 | 27.5±0.3 | 23.6±0.7 | <0.001 |
| **APOE4 carriers (%)** | 39% | 50% | 84% | 70% |  |
| **Alb_CSF_/Alb_serum_** | 5.9±0.4 | 6.6±0.6 | 5.5±0.8 | 6.8±0.5 |  |

Data are represented as mean ± standard deviation. Education and age in years. MMSE = Mini Mental State Examination. Alb= albumin.

The patients included in this study (n=99) were from the Memory Clinic at the Karolinska University Hospital in Huddinge, Sweden. The groups selected were similar to those in the ADNI study in age, MMSE score and AD CSF biomarkers.These patients were all living independently in the community. They were evaluated according to a standard comprehensive assessment protocol including clinical examination, brain imaging, electroencephalography, analyses of blood (serum albumin, glucose) and CSF (including albumin, total tau (T-Tau), phospho-tau (P-Tau), and Aβ_1-42_) and a detailed neuropsychological evaluation. Dementia and AD were diagnosed according to DSM-IV and NINCDS-ADRDA criteria. MCI patients were (1) not demented, (2) had (self and/or an informant) reported cognitive decline and impairment on objective cognitive tasks, and (3) had preserved basic ADL/minimal impairment in complex instrumental functions. Patients with psychiatric disorders (i.e. depression, alcohol abuse) or other conditions (i.e. diabetes, brain tumors, normal pressure hydrocephalus) were not included. The study was conducted under the guidelines of the Declaration of Helsinki and approved by the ethics committee of the Karolinska Institutet.
